# Study Protocol: Southern Women Veterans’ Health Study

**Published:** 2018-01-18

**Authors:** Steven S Coughlin, Kimberly Sullivan

**Affiliations:** 1 Department of Clinical and Digital Health Sciences, Augusta University, Augusta, GA, USA; 2 School of Public Health, Boston University, Boston, MA, USA

**Keywords:** Epidemiologic studies, Veterans, Women

## Abstract

**Introduction:**

Despite the large numbers of women who served during the 1990–1991 Gulf War or post 9/11, many studies of the health of U.S. veterans have been limited to men or have not included adequate numbers of women veterans for separate analyses. As the number of women in the military increases particularly in the Southern U.S., there is a pressing need for additional health research involving women veterans including women who reside in rural areas and small towns and cities. Studies are also needed of women who do or do not receive VA health care services.

**Objectives:**

The objectives of this study are: 1) To obtain information about the physical and mental health of women who served in the U.S. military during various eras (e.g., Vietnam War, 1991 Gulf War, and post 9/11) and who currently reside in Georgia, 2) To establish a cohort of women veterans who complete a baseline postal questionnaire and are then followed longitudinally and invited to participate in a follow-up survey in 4 to 5 years, and 3) To establish a cohort of women veterans who can be invited to participate in IRB-approved clinical research studies that include subsets of women surveyed as part of the Southern Women Veteran’s Health Study.

**Methods:**

This study consists of a postal survey of up to 1,000 women veterans in Georgia and a repeat survey in 4 to 5 years to obtain longitudinal data. The follow-up survey in 4 to 5 years will allow for longitudinal changes in health to be assessed.

**Conclusion:**

The survey will provide a comprehensive picture of women veteran’s health across the lifespan. This will address the need for a comprehensive surveillance of health outcomes of women veterans with diverse residence (rural areas, larger cities in the southern U.S.) and VA health care service utilization. A broad range of women’s health issues will be addressed including not only reproductive outcomes but also chronic health outcomes in endocrine, cardiac, neurological, immunological and mental health systems that may differ from their male counterparts. Through its longitudinal design, the study will also provide important information about changes in physical and mental health as women veterans advance in age.

## Introduction

There are about 1.8 million living women veterans in the U.S. [1]. Although women have played a role in the U.S. military since 1775 (for example, as nurses and cooks), it was not until 1948 that a law was passed that made women a permanent part of the military services [2]. Since then, women have increasingly served in all branches of the military, and in both active and reserve components.

During 1990–1991, seven percent or about 40,000 U.S. military women served during the Gulf War operations Desert Shield and Desert Storm [3]. Until 2013, women were prohibited from being assigned to ground combat units below the brigade level. According to statistics from 2013, 15.6 percent of the U.S. Army’s 1.1 million soldiers, including National Guard and Reserve, were female. That year, women served in 95% of all Army occupations. Thus, the number of women serving has doubled and the occupational tasks have increased exponentially necessitating a more careful review and monitoring of women military veterans’ health.

Women are impacted by military service and deployment differently than men, underscoring the need for studies of the health of women veterans and their receipt of health care services. In 2008, 453,250 women veterans were enrolled in the VA health care system. The number of women veterans enrolled in the VA health care system doubled over the past decade because of substantial numbers of veterans returning from Operation Enduring Freedom, Operation Iraqi Freedom, and Operation New Dawn (OEF/OIF/OND) [4]. Post-traumatic stress disorder (PTSD), depression, and hypertension are among the top diagnostic categories for women veterans treated by VA health care. Military sexual trauma contributes to elevated PTSD rates among women veterans as well [5].

Many women veterans suffer from chronic pain (migraines, other types of headaches, and pain in the lumbosacral spine or lower extremities), diabetes, cardiovascular disease, and asthma [6–8]. Traumatic brain injury, PTSD, depression, insomnia, and chronic pain are becoming more important causes of morbidity among women veterans as women take on combat roles during their military service. In a survey of women veterans who received health care at VA Greater Los Angeles Health-care System, the prevalence of insomnia was 52.3% [9]. In 2014, 44% of women receiving VA health care were obese and 31% were overweight, which is comparable to rates in the general U.S. population [10]. About 68% of women veterans with diabetes were obese [10]. During 2011–2013, an estimated 31.3% of women veterans had arthritis [11]. Many women veterans also suffer from symptom-based health conditions such as medically unexplained chronic multisymptom illness, Gulf War Illness, fibromyalgia, and chronic fatigue syndrome [3,12,13].

About 12% of women veterans who use VA health care, including women who served during the Vietnam War and Korean War, are >65 years. Based upon data from the National Survey of Women Veterans conducted in 2008–2009, almost 70% of women veterans >65 years have three or more comorbid chronic conditions such as arthritis, hypertension, depression, chronic pulmonary disease, cancer, and post-traumatic stress disorder [14].

Despite the large numbers of women who served during the 1990–1991 Gulf War or post 9/11 (OEF/OIF/OND), many studies of the health of U.S. veterans have been limited to men or have not included adequate numbers of women veterans for separate analyses [4,15]. Some studies have been limited to women receiving VA health care services in large urban areas such as Los Angeles [9]. Other studies such as the Women Veterans Cohort Study and the Million Veteran Program do not include veterans who do not receive VA health care services. There is a pressing need for additional health research involving women veterans who reside in the southern U.S., including women who reside in rural areas and small towns and cities, and women who do or do not receive VA health care services.

The objectives of this study are: 1) To obtain information about the physical and mental health of women who served in the U.S. military and who reside in Georgia, 2) To establish a cohort of women veterans who complete a baseline postal questionnaire and are then followed longitudinally and invited to participate in a follow-up survey in 4 to 5 years, and 3) To establish a cohort of women veterans who can be invited to participate in Institutional Review Board **(IRB)**-approved clinical research studies that include subsets of women surveyed as part of the Southern Women Veterans’ Health Study.

## Materials and methods

This study consists of a postal survey and a repeat survey in 4 to 5 years to obtain longitudinal data. The follow-up survey in 4 to 5 years will allow for longitudinal changes in health to be assessed.

## Study Population

The study population will consist of up to 1,000 women veterans who currently reside in Georgia. Women who served in different eras (Korean War, Vietnam War, 1990–1991 Gulf War, OEF/OIF/OND) will be included so that it will be possible to study women veteran’s health across the lifespan. Non-institutionalized women veterans will be eligible to take part in this study if they reside in Georgia and are able to provide informed consent to a postal survey. In addition to having a large veteran population, the State of Georgia is home of several US military installations including Fort Gordon, Fort Stewart, Fort Benning, Moody Air Force Base, Robins Air Force Base, Naval Submarine Base Kings Bay, Marine Corps Logistics Base Albany, Coast Guard Air Station Savannah, and Coast Guard Station Brunswick.

## Data Collection

Data will be collected using postal survey questionnaires developed in a Teleform optical character recognition system. All subjects will have an assigned a participant ID number. A sequential mailing protocol will be followed using a modified Dill-man method. An advance letter will be mailed to the women by the study principal investigator. The letter will provide information about the study (purpose, potential benefits, and risks) and let them know how they can opt out and not receive further mailings about the study. Three weeks later, a survey consent letter will be mailed to women who have not opted out along with a copy of the survey questionnaire and a pre-addressed, stamped return envelope. The baseline and follow-up survey questionnaires will take about 30 to 45 minutes to complete.

Women who have not opted out or returned a completed questionnaire will be sent a reminder postcard 3 weeks later, followed 3 weeks later by a duplicate copy of the survey consent letter, survey questionnaire, and a pre-addressed, stamped return envelope. Women who have not opted out or returned a completed questionnaire will be sent a second reminder post card 3 weeks later. In about 4 to 5 years, the panel of women who participate in the baseline survey will be re-contacted for a follow-up postal survey or telephone interview. Survey responses will be checked for completeness and then coded and scanned using Teleform software for entry into an electronic database. All survey data collected as part of the study will be carefully monitored for completeness. If a veteran returns two copies of the questionnaire, the most complete questionnaire will be selected for inclusion. The quality of the data will be maximized through pre-coded responses and computerized internal consistency checks and range checks of specified values. The use of Teleform software for scanning paper questionnaires will maximize the accuracy of computer databases.

The data collected in this way will be used to examine the prevalence and patterns of symptoms, diagnosed medical conditions, reproductive health, birth outcomes, and other health issues among women who served during the Gulf War. Statistical analyses will focus on health outcomes that affect women veterans who served during different eras (e.g., Vietnam, 1990–1991 Gulf War, OEF/OIF/OND). The follow-up survey in 4 to 5 years will allow for longitudinal changes in health to be assessed.

## Advisory Committee

In order to help ensure the success of the study, a Women Veterans’ Advisory Committee has been established that includes Ms. Veda Brooks, the Women’s Veterans Coordinator for the Georgia Department of Veterans Services in Atlanta; Wanda Jirau-Rosaly, MD, a geriatrician in the Division of General Internal Medicine at the Medical College of Georgia; a women veteran who served in the U.S. Army during the 1990–1991 Gulf War era and who resides in the Atlanta area; and two women veterans who were referred by the Director of Military and Veterans Services at Augusta University. The Advisory Committee will hold 3 to 4 conference telephone calls per year. The role of the Advisory Committee will be to provide recommendations about how best to let women veteran’s in Georgia know about the study (e.g., news items in Georgia Department of Veterans Service announcement, American Legion newsletters, or media release prepared by Augusta University Communications) and how best to disseminate findings from the study to women veterans in our state. The Advisory Committee will be chaired by Ms. Veda Brooks who is not only Women Veterans Coordinator for the Georgia Department of Veterans Services but also active in women services for the American Legion in Georgia.

## Recruitment

Women veterans’ names and mailing addresses were obtained from a commercial firm (Lake Group Media, Armonk, NY). The name and address of each veteran (n=19,477) was known because they participated in a telemarketing survey or had a deed transfer posted in County Courthouse Public Records. Only women veterans who reside in Georgia are included in the computer file. All residents of Augusta-Richmond County who live in close proximity to the Augusta University Health Sciences Center where clinical research sub-studies may be conducted, will be included in the initial mailings. In order to obtain a sample of up to 1,000 completed survey questionnaires, random numbers were generated from 0 to 19,477, with no repeat numbers, and assigned to each individual in the file. A random sample of women veterans who reside in Georgia outside of Augusta-Richmond County will then be obtained in this way. In the first stage of the postal survey, the mailings will be sent to 1,000 potential research participants (residents of Augusta-Richmond County plus additional women veterans randomly sampled from potential research participants who live elsewhere in Georgia). Because the response rate to the postal survey is likely to be in the range of 40–60%, it will be necessary to obtain an additional random sample of women veterans included in the computer file of names and mailing addresses in the second phase of the postal survey so that the final study population is about up to 1,000 participants.

## Data Analyses

The approach that will be taken for statistical analysis of the data is as follows. Initially, crosstabulations of the data will be performed using SAS. Both chi-square and Fisher’s exact tests will be used to examine the statistical significance of observed associations. After crosstabulations and exploratory analyses of the survey data are completed, logistic regression methods will be used to compare groups of women veterans with respect to frequency of symptoms, medical conditions, and exposures. Potential confounding factors will be controlled for in these analyses (e.g., age, military rank). Potential effect modifiers will initially be examined in exploratory crosstabulations of the data and then by including interaction terms in logistic models and performing Log-likelihood ratio tests). Ninety-five percent confidence intervals will be obtained for adjusted odds ratios. Levels of statistical significance will be determined using Wald chi-square tests and Log-likelihood ratio tests. The goodness-offit of each model will be examined using the Log-likelihood ratio tests. Potential confounding will be dealt with through stratification and by controlling for study cohort in logistic regression models.

To analyze data on the frequency and patterns of veteran reported chronic symptoms and medical conditions diagnosed by healthcare providers, crosstabulations of the data will be performed. Chi-square and Fisher’s exact tests will be used to examine the statistical significance of observed associations. Prevalence odds ratios will be obtained with 95% confidence intervals. Additional analyses will be stratified on branch of service, component, or study cohort. Prevalence odds ratios associated with chronic symptoms and medical conditions will be obtained using logistic regression. These outcomes will be assessed in relation to military, deployment, and demographic characteristics using stratified analyses and included in multivariable models as appropriate. Military characteristics (era of military service, branch of service, active or reserve component, deployment status, rank) and demographic variables (age category, sex, and education) will be included in the models.

Variables included in the analysis will be supplemented with two county-level variables. A variable for rural, suburban, or urban county of residence will be obtained using the research participant’s zip code and rural-urban continuum codes obtained from the U.S. Department of Agriculture [16]. This will allow for health care utilization to be examined according to whether the veteran lives in a rural, suburban, or urban county. A variable for whether or not the county of residence has been designated by the Health Resource and Services Administration **(HRSA)** as a health professional shortage area will be obtained from the HRSA Area Resource File [17].

Analyses of general health and functional status, use of healthcare services, and hospitalizations will consist of descriptive analyses (frequency distributions and crosstabulations) of the data performed using SAS. Additional analyses will be stratified by era of military service and deployment status. Both chi-square and Fisher’s exact tests will be used to examine the statistical significance of observed associations. Utilization of healthcare services will be examined by two county characteristics (rural/suburban/urban and healthcare professional shortage area).

Analyses of veteran-reported pregnancy and birth outcomes among women veterans will also consist of frequency distributions and crosstabulations of the data, stratified by deployment status. Additional analyses will be stratified on era of military service, deployment status, branch of service, or component. The Mantel-Haenzel procedure and logistic regression methods will be used to adjust for age and other potential confounding variables.

Changes in women veterans’ health over time will be examined using baseline and follow-up data. These analyses will be limited to women who participated in both the baseline and follow-up surveys. Crosstabulations of the data will initially be performed. Chi-square and Fisher’s exact tests will be used to examine the statistical significance of observed temporal associations. Prevalence odds ratios associated with chronic symptoms and chronic medical conditions will be obtained using logistic regression. Military characteristics (era of military service, branch of service, component, deployment status, rank) and demographic variables (age at baseline, education) will be included in the models as appropriate. Additional comparisons will be made with data from the National Health and Nutrition Examination Survey.

## Sample Size Calculations

Sample size calculations were carried out based upon an array of assumptions, taking into account likely attrition. Based upon a literature review, the percentage of women who report a history of medical conditions of interest will likely vary widely between 5–50%, depending upon the current prevalence of common chronic conditions such as post-traumatic stress disorder, migraine headaches, diabetes, and hypertension. Sample size calculations were carried out for a two-tailed test on proportions (P1, P2). For a two-tailed test on proportions, with a type I error rate of 5% and power of .80, and where P1 = .15 and P2=.30, 134 women per group (or 268 women total) would be needed to detect this difference. For ordinal variables, the statistical power to detect meaningful differences across groups will be greater.

## Limitations

Although validation studies have shown that veterans are able to accurately report information about medical conditions and health care utilization [18,19], information obtained about deployment-related exposures that occurred during military service in the distant past is subject to misclassification bias. Because one of the goals of the Southern Women Veteran’s Health Study is to establish a cohort of women veterans who can be invited to participate in clinical research studies, it may be possible to validate some medical conditions and exposures at the time of these sub-studies.

## Women Veterans’ Advisory Committee

To ensure the success of the study, a Women Veterans’ Advisory Committee has been established that is chaired by the Women Veterans Coordinator for the Georgia Department of Veterans Services in Atlanta. The Advisory Committee will hold 3 to 4 conference telephone calls per year. The role of the Advisory Committee is to provide recommendations about how best to let women veterans in Georgia know about the study (e.g., news items in Georgia Department of Veterans Service announcement, American Legion newsletters, or media release prepared by Augusta University Communications) and how best to disseminate findings from the study to women veterans.

## Human Subjects

The study protocol has been approved by the Augusta University IRB. There are no known risks to participants in this survey other than potential, minor psychological distress. The potential benefits of the study are societal in nature and include obtaining new information about the health and health care utilization of women veterans. There is no direct benefit to participants in this study. Personal identifiers will be removed from study-related health information and kept in a locking cabinet in the principal investigator’s office at Augusta University. Paper-based records will be kept under lock and key and will only be accessible to personnel involved with this study. Data files will be kept on password protected office computers at Augusta University and the Boston University Data Coordinating Center. Only members of the study team will have access to the data.

## Conclusion

The Southern Women Veterans’ Health Study will address the need for information about the comprehensive health of women veterans with diverse residence (rural areas, small towns, larger cities in the southern U.S.) and VA health care service utilization. The study is likely to contribute importantly to our understanding of key women veteran’s health concerns across the lifespan from young adulthood to older age. A broad range of women’s health issues will be addressed including adverse reproductive outcomes as well as rates of GWI, endocrine, cardiac, neurological, immune and psychological disorders. Through its longitudinal design, the study will provide important information about changes in physical and mental health as women veterans’ age. This includes the health of women veterans who served in the military decades ago (e.g., Vietnam veterans) and those who are now facing chronic conditions that are prevalent among older persons. A particular goal of the study is to establish a cohort of women veterans who can be invited to participate in IRB-approved clinical research studies that include subsets of women surveyed as part of the Southern Women Veterans’ Health Study. Potential topics for these sub-studies include symptom-based conditions such as Gulf War illness and chronic fatigue syndrome; chronic pain, headache, traumatic brain injury, and other neurological conditions; and chronic conditions that are prevalent in both veteran and non-veteran populations as people reach middle age or older age (for example, diabetes, cardiovascular disease, rheumatoid arthritis, chronic pulmonary conditions).

There is a strong need for studies with well-established cohorts of veterans, particularly in under-represented groups including women veterans who are rapidly becoming a much larger part of the total military personnel. This requires that their health should be more fully characterized for unique health outcomes across the lifespan to improve military readiness while serving and for appropriate health care planning of VA women’s health clinics.
